# Habitat amount or landscape configuration: Emerging HotSpot analysis reveals the importance of habitat amount for a grassland bird in South Dakota

**DOI:** 10.1371/journal.pone.0274808

**Published:** 2022-09-26

**Authors:** Sprih Harsh, Robert C. Lonsinger, Andrew J. Gregory

**Affiliations:** 1 Department of Natural Resource Management, South Dakota State University, Brookings, South Dakota, United States of America; 2 Department of Biological Science, University of North Texas, Texas, United States of America; Feroze Gandhi Degree College, INDIA

## Abstract

Habitat loss and fragmentation are two important drivers of biodiversity decline. Understanding how species respond to landscape composition and configuration in dynamic landscapes is of great importance for informing the conservation and management of grassland species. With limited conservation resources, prescribed management targeted at the appropriate landscape process is necessary for the effective management of species. We used pheasants (*Phasianus colchicus*) across South Dakota, USA as a model species to identify environmental factors driving spatiotemporal variation in population productivity. Using an emerging Hotspot analysis, we analyzed annual count data from 105 fixed pheasant brood routes over a 24-year period to identify high (HotSpot) and low (ColdSpot) pheasant population productivity areas. We then applied classification and regression tree modeling to evaluate landscape attributes associated with pheasant productivity among spatial scales (500 m and 1000 m). We found that the amount of grassland at a local spatial scale was the primary factor influencing an area being a HotSpot. Our results also demonstrated non-significant or weak effects of fragmentation *per se* on pheasant populations. These findings are in accordance with the habitat amount hypothesis highlighting the importance of habitat amount in the landscape for maintaining and increasing the pheasant population. We, therefore, recommend that managers should focus on increasing the total habitat area in the landscape and restoring degraded habitats. Our method of identifying areas of high productivity across the landscape can be applied to other species with count data.

## Introduction

Habitat loss and fragmentation are two of the greatest threats to wildlife conservation [1,2]. The fragmentation process involves the splitting of natural habitat into smaller, more isolated patches and is intrinsically coupled with habitat loss [3]. While habitat loss can lead to a decline in wildlife populations, habitat fragmentation may increase the cost of moving among habitat patches and therefore reduce the accessibility and suitability of surrounding patches for wildlife [3]. Habitat loss and fragmentation combined with greater exposure to human land uses have resulted in widespread declines in biodiversity. These landscape changes have been linked to negative impacts on populations of fish [4], mammals [5], birds [6,7], insects [8], and plants [9]. Indeed, one of the key questions in conservation biology is determining the effects of habitat loss versus habitat fragmentation *per se* [10,11]. This further leads to the debate of conserving multiple small or fewer large habitat patches [12].

Grasslands are among the most threatened biomes worldwide [13]. In North America, nearly 98% of the native northern tallgrass prairie has been lost to the cultivation of row crops and the planting of non-native grasses for livestock production [14]. Numerous species have suffered severe population declines as a result of the frequency and intensity of landscape changes. Grassland songbirds are experiencing the steepest population decline of any bird group [15]. From 1968 to 2008, 37% of grassland obligate bird species experienced a population decline [16]. In the United States, South Dakota has experienced a substantial decline in perennial grassland [17,18]. Between 2006 and 2012 South Dakota lost ~76% of total extant grassland areas to other land uses [19] which has threatened its grassland species.

The ring-necked pheasant (*Phasianus colchicus;* hereafter pheasant) is an edge-tolerant species that is negatively impacted by the conversion of grassland to cultivation [20,21]. Pheasants were introduced to the United States in the early 1900s and they soon adapted to not only coexist but thrive with primitive agriculture [22]. The landscapes at that time were high-quality pheasant habitats. Relatively primitive agricultural practices created a landscape containing a diversity of crop types established over a variety of field sizes [22,23]. Abundant weeds in the crop fields and inefficient harvest of grain leaving waste grains helped provide ideal brood habitat and high-quality winter cover [22]. For the past 30 years, however, cultivation has intensified leading to a decline in grassland and emergent wetland area or habitat quality [22,23]. In South Dakota, nearly 58% of grassland loss from 2006–2012 occurred in key pheasant regions [19], and pheasant populations have been declining since then [22]. For example, annual brood survey data in South Dakota indicated a nearly 41% decline in pheasant relative abundance from 2008 to 2018 [24], which coincided with a 37% reduction in the area of grasslands enrolled in the Conservation Reserve Program and a 24% increase in the area of harvestable corn and soybean [25]. Although introduced, pheasants are economically and socially important in South Dakota. According to the South Dakota Department of Game, Fish, and Parks [26], pheasants are the most sought-after and profitable upland game species, with pheasant harvest being a multimillion-dollar industry in the state. Pheasant hunting is also an important social activity that reunites families and friends [26]. A recent analysis suggested that in a single county in South Dakota, pheasant hunting generated $9.7 million in economic benefit and created 111 jobs (Gregory and Mills, *unpublished data*).

Pheasants are important to the economy and culture of South Dakota [26] and, therefore, conserving pheasants can protect the habitat for native grassland species. By attracting funding from individual donors, and wildlife organizations, they act as a surrogate for broader biodiversity conservation, especially grassland species. The distribution, abundance, and survival of this species reflect the quality and conservation status of the grassland it inhabits. Understanding the drivers of recent broad-scale pheasant population declines in South Dakota is an important management objective and can provide insights into the sensitivity of a grassland species to landscape changes. Moreover, the dynamic nature of this agriculturally dominated landscape provides an opportunity to investigate species-habitat relationships and identify landscape attributes useful in predicting habitat quality. Furthermore, habitat loss or habitat fragmentation does not affect all species equally; sensitivity to these processes varies with species’ numerous ecological traits. We, therefore, need to understand how the habitat amount and configurational landscape heterogeneity (connectivity of fragments, number of fragments) influence this species’ abundance. With limited conservation resources, targeted and prescribed management at the appropriate landscape process at the appropriate spatial scale is required to optimize conservation efforts. Here, we aim to identify landscape factors, whether habitat amount or habitat configuration, influencing spatiotemporal variation in pheasant productivity across South Dakota.

In this study, we used an emerging HotSpot analysis of annual pheasant brood survey data to investigate the spatial and temporal drivers of pheasant population dynamics. Specifically, we evaluated 1) the spatial and temporal variability of high and low pheasant productivity areas in South Dakota, 2) the spatial context and landscape heterogeneity of high pheasant productivity areas to areas under agricultural production, 3) the degree to which high pheasant productivity areas were correlated to natural land cover, and 4) how the inter-juxtaposition of agricultural land uses, and natural areas impacted pheasant productivity.

## Methods

### Study system

South Dakota is part of the prairie potholes ecosystem and is comprised primarily of open grasslands east of the Missouri River and upland steppe ecotypes in the west. Our study occurred primarily in eastern South Dakota, which was characterized by tallgrass prairie and highly fragmented by agriculture [27,28]. Our study system had a mid-continent mid-latitude temperature and precipitation regime characterized by cold snowy winters and hot dry summers. The average low temperature for January was ~11°C, while the average high temperature for July was ~30°C. Late springs and early summers experienced moderate rainfall with average annual precipitation of 508 mm [29]. Cultivated agriculture was the dominant land use and a key component of the regional economy, accounting for nearly $25.6 billion (~30%) of South Dakota’s total economy [30].

### Pheasant data

We used annual pheasant brood survey data collected from 1993 to 2016 by the South Dakota Department of Game, Fish, and Parks. Annual pheasant brood surveys included counts of males, females, and broods observed along 110 fixed 48-km survey routes distributed across the pheasant range in South Dakota [31]. Routes were surveyed from 25 July to 15 August each year using standardized methods on mornings when weather conditions were optimal for detecting pheasants (i.e., clear skies, heavy dew, and light winds). During surveys, one observer counted the number of pheasants and broods observed within 0.2 km of the roadway while driving at a speed <48 km/hour [31]. Raw pheasant counts were converted into a pheasant*km^-1^ index of pheasant abundance [24].

**Fig 1:**
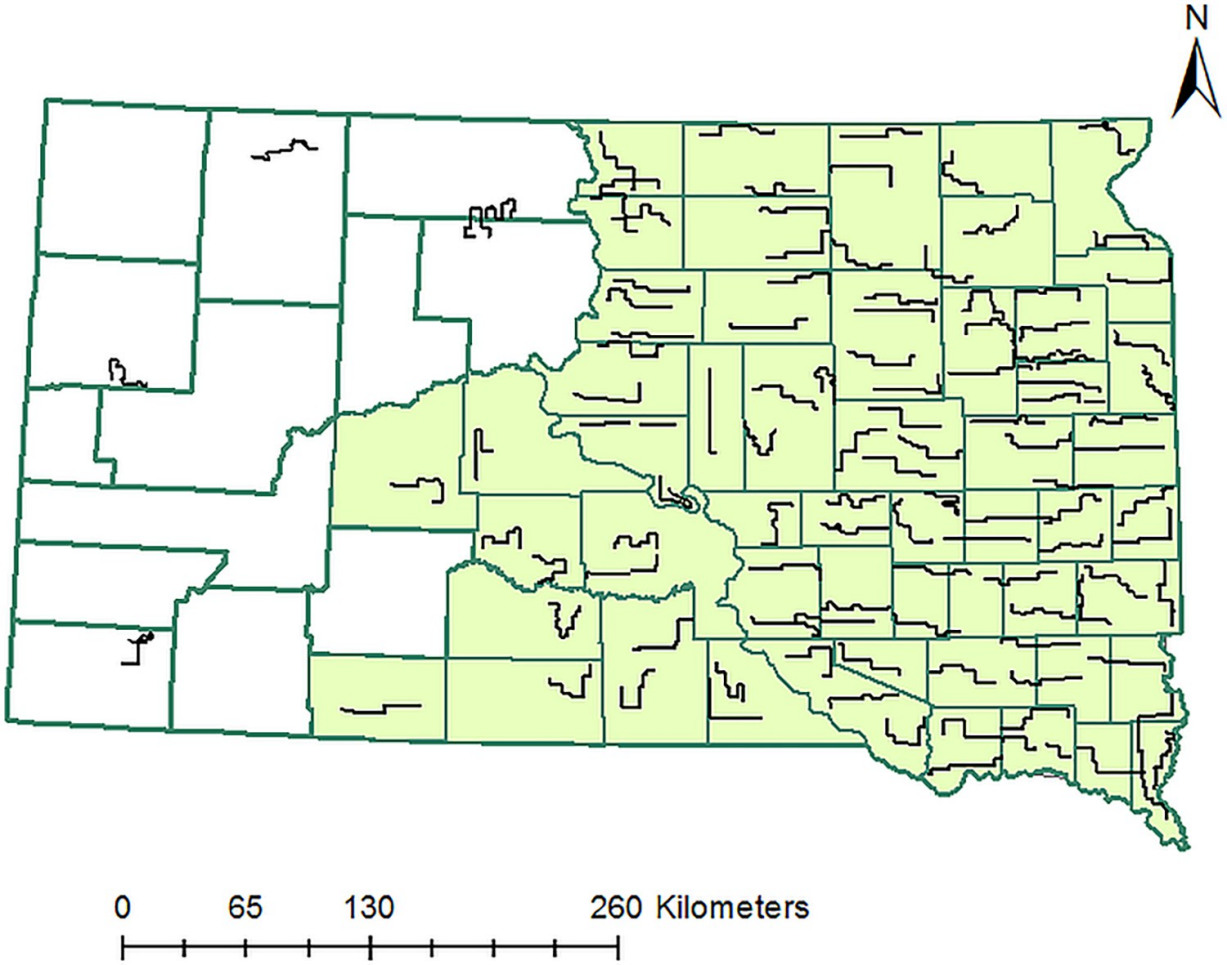
Distribution of roadside survey routes used to index the relative abundance of pheasants in South Dakota; data collected along the 105 survey routes within the highlighted 53 counties were used to assess factors driving pheasant productivity between 1993–2016.

We censored 5 routes that were west of the Missouri River where route density was too low to adequately parameterize the spatial analysis and account for the difference in land cover (i.e., dominated by mixed-grass prairie) from the tallgrass prairies (Fig 1). Spatial coverage of the remaining 105 routes (93 located east and 12 southwest of the Missouri River) aligned with areas where pheasant populations in South Dakota were concentrated; thus, our sampling extent included the majority of the pheasant population in South Dakota (Fig 1, [31]).

### Land cover data

We used the Cropland Data Layer (CDL) to characterize the land cover for each route [32]. One drawback to the CDL for South Dakota is that data is not available before 2006 [33]. Therefore, we confined our analysis of the influence of land cover to the 11-year period from 2006 to 2016. We reclassified the original 133 CDL land-cover classes into five cover classes: grassland, row crops, small grains, wetlands, and others. Grass-dominated land cover ranged from native prairie to anthropogenically altered grasslands such as hay lands and pastures. Because of their spectral similarity, these different cover types were difficult to resolve in satellite imagery. Agricultural crops including corn, soybeans, and sorghum were categorized as row crops. Crops including wheat, barley, and oats were classified as small grains. Woody and herbaceous wetlands were classified as wetlands. The remaining land-cover types were classified as others [34].

### Identifying areas of high, average, and low pheasant productivity

To identify areas as high (HotSpots), average (AverageSpots), and low (ColdSpots) pheasant productivity, all routes were converted to point features using ArcGIS version 10.6 (ESRI, Inc., Redlands, CA, USA) where each point depicted the mid-point of the respective route. We then applied the Getis-Ord GI* statistic to conduct an independent HotSpot analysis of pheasant*km^-1^ for each year from 1993 to 2016 [35]. We used incremental spatial autocorrelation to identify the distance band threshold that exhibited maximum clustering [36]. Once we determined HotSpots, AverageSpots, and ColdSpots for each year of the 24-year study period, we created separate point feature files for significant HotSpots and ColdSpots stratified by year, and then bound those areas using a minimum convex polygon (MCP). This yielded a set of 24 HotSpot raster images and a set of 24 ColdSpot raster images, one for each year. In each of these HotSpot or ColdSpot MCPs, we coded 1 for those areas that were HotSpots or ColdSpots, respectively, and 0 for all others. We then overlaid the HotSpot or ColdSpot MCP layers and summed the MCPs to calculate the number of times over our 24-year study period that an area was a HotSpot or ColdSpot. Similarly, we calculated areas that were AverageSpots. To characterize the trend in pheasant population across these different levels of pheasant productivity, we then calculated average pheasant*km^-1^ along routes identified as HotSpots, ColdSpots, and AverageSpots.

### Determination of landscape characteristics

We computed landscape metrics associated with our reclassified land-cover data for each route classified as either a HotSpot or as a ColdSpot annually for the 11-year period from 2006 to 2016 with FRAGSTATS version 4.2 [37]. All landscape metrics from FRAGSTATS were computed at two spatial neighborhoods (500 m and 1000 m). This process involved the creation of 500-m and 1000-m buffers around each route (1 and 2 times the average pheasant home range size during nesting and brooding seasons, respectively; [38,39]). We chose routes, instead of points, for creating buffers because landscape characteristics across these 48-km routes describe an area being a HotSpot or a ColdSpot and allow us to link spatial land cover land use attributes associated with that area which may influence that area being a HotSpot or ColdSpot. Reclassified land cover was then extracted for each of the buffered routes and used to calculate landscape metrics that we predicted would be important factors influencing pheasant HotSpots based on the ecology of gallinaceous birds. This included composition, contiguity, and fragmentation metrics of each land-cover class for each spatial neighborhood [40–42]. Composition metrics included the proportion of area of each land-cover type in each buffer. The contiguity of land cover was measured using the contiguity index, which represented the size and connectivity of patches of a given land-cover type on a scale of 0 (small patches) to 1 (large and contiguous patches). Fragmentation was measured using the number of patches, which summed the number of patches of each land-cover type at each scale. Increasing fragmentation of a land-cover type represented an increase in the number of patches. We also assumed that pheasant HotSpots would be impacted by the total number of patches in the landscape, and contiguity of the landscape at both 500-m and 1000-m scales.

### Data analysis

The analysis of pheasant HotSpots was conducted using a classification and regression tree (CART, [43]) approach. CART is a nonparametric machine learning algorithm that makes no assumptions about relationships between features and is robust to correlated variables [44]. The CART analysis was conducted using the *rpart* package in the R programming language [45]. To begin the CART analysis, simple random sampling without replacement was used to partition the full data set into a training dataset containing 80% of the observations and a testing and validation data set containing 20% of the observations. A CART was then parameterized with all independent variables at both 500-m and 1000-m scales. The CART was applied first on the training data set and then on the test data set to assess the model generalizability and to evaluate any over-fitting of the model to the training sample. A confusion error matrix was then used to further evaluate model performance [46].

## Results

**Fig 2:**
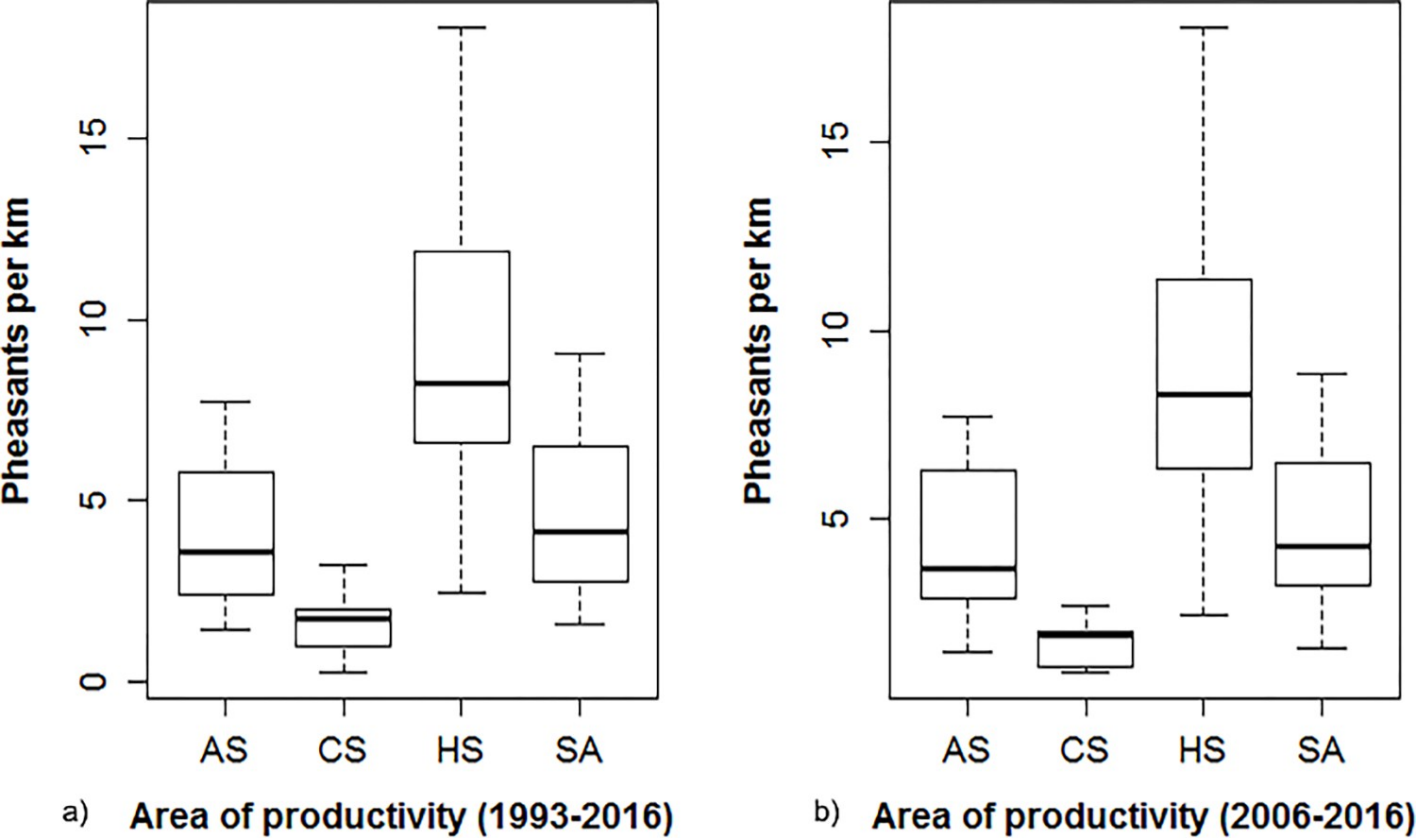
Boxplots of pheasants*km^-1^ over a) 24-year and b) 11-year period across the study area (SA), HotSpot (HS), ColdSpot (CS) and AverageSpot (AS).

**Fig 3:**
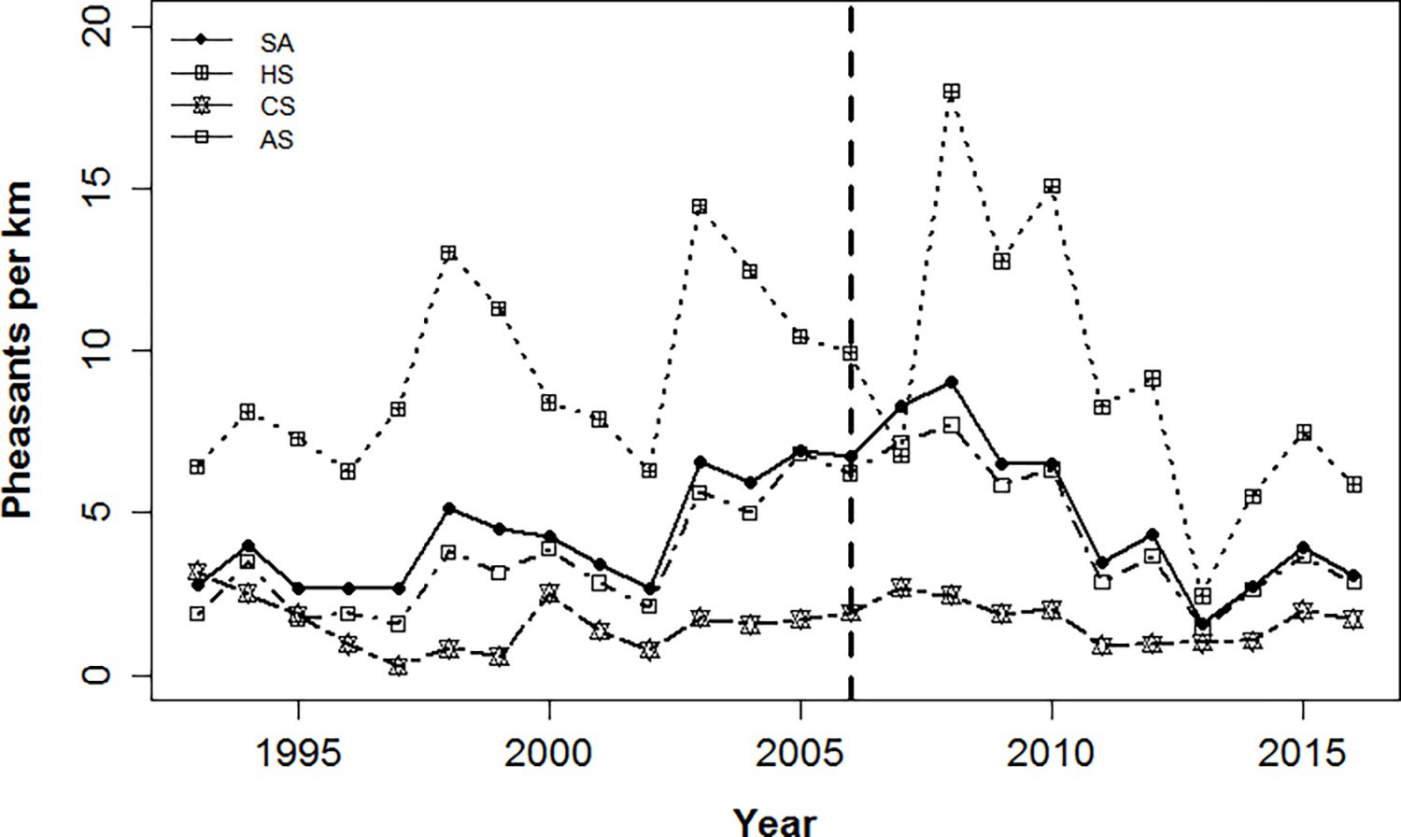
Trend in pheasants*km^-1^ over 24-year and 11-year (after vertical line) period across the study area (SA), HotSpot (HS), ColdSpot (CS) and AverageSpot (AS).

Of 105 brood routes included in the analysis, 54 routes contributed towards the creation of HotSpots in ≥1 year, 36 routes were part of ColdSpots in ≥1 year, and 99 routes were part of AverageSpots in ≥1 year. Over the 24-year period, the average count of pheasant*km^-1^ was 4.60 ± 0.41 in the study area, 3.94 ± 0.89 for AverageSpots, 9.26 ± 0.74 for HotSpots, and 1.61 ± 0.16 for ColdSpots (Fig 2A). During this period study area, and AverageSpots demonstrated a positive population trend (0.04 ± 0.06 pheasant*km^-1^*year^-1^ and 0.9 ± 0.75 pheasant*km^-1^*year^-1^, respectively; Fig 3) while HotSpots and ColdSpots exhibited a negative trend (−0.06 ± 0.11 pheasant*km^-1^*year^-1^ and −0.79 ± 0.02 pheasant*km^-1^*year^-1^, respectively; Fig 3). Although the trend in pheasant indices over the 24-year period was positive (0.04 ± 0.06 pheasant*km^-1^*year^-1^), there was high variation in the number of pheasants per km suggesting that the population may have been relatively stable across the longer 24-year period. During the course of 11-year period, the average count of pheasant*km^-1^ was 4.89 ± 0.68 in the study area, 4.59 ± 0.99 for AverageSpots, 9.22 ± 0.32 for HotSpots, and 1.69 ± 0.18 for ColdSpots (Fig 2B). This 11-year period showed negative population trend across all levels of pheasant productivity (study area: −0.59 ± 0.13 pheasant*km^-1^*year^-1^, AverageSpots: −2.81 ± 0.49 pheasant*km^-1^*year^-1^, HotSpots: −0.39 ± 0.37 pheasant*km^-1^*year^-1^, and ColdSpots: −1.27 ± 0.29 pheasants*km^-1^*yr^-1^; Fig 3).

**Fig 4:**
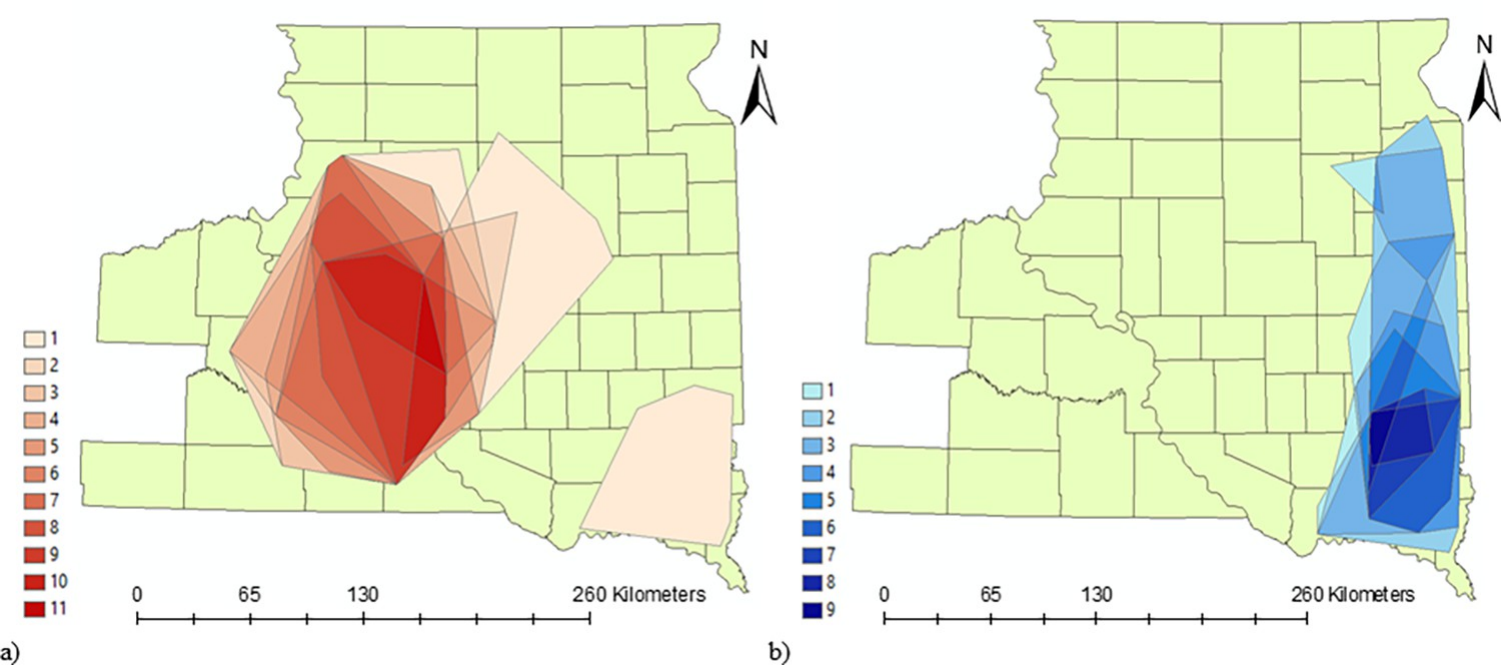
Areas under a) HotSpot and b) ColdSpot over an 11-year (2006–2016) period across the study area in South Dakota, USA. The legend shows the number of years an area was either a HotSpot or a ColdSpot.

The total area that was a HotSpot for some period of time during the 11-year study period was 47,643 km^2^ (~38% of the total study area). This included a core area of 3,512 km^2^ that was consistently a HotSpot for all 11 years (Fig 4A). ColdSpots occupied a total of 20,846 km^2^ (~17% of the study area), of which, there was a 454 km^2^ core that was a ColdSpot for 9 of 11 years (Fig 4B. The maximum number of years that an area was a ColdSpot was 9 of 11 years as there was no overlap of ColdSpots from 2015 or 2016. HotSpots over the 24-year period had an additional area of 7,034 km^2^ compared to HotSpots over the 11-year period. Similarly, ColdSpots for the 24-year period had an additional area of 3,938 km^2^ compared to ColdSpots for the 11-year period (S1 File).

**Fig 5:**
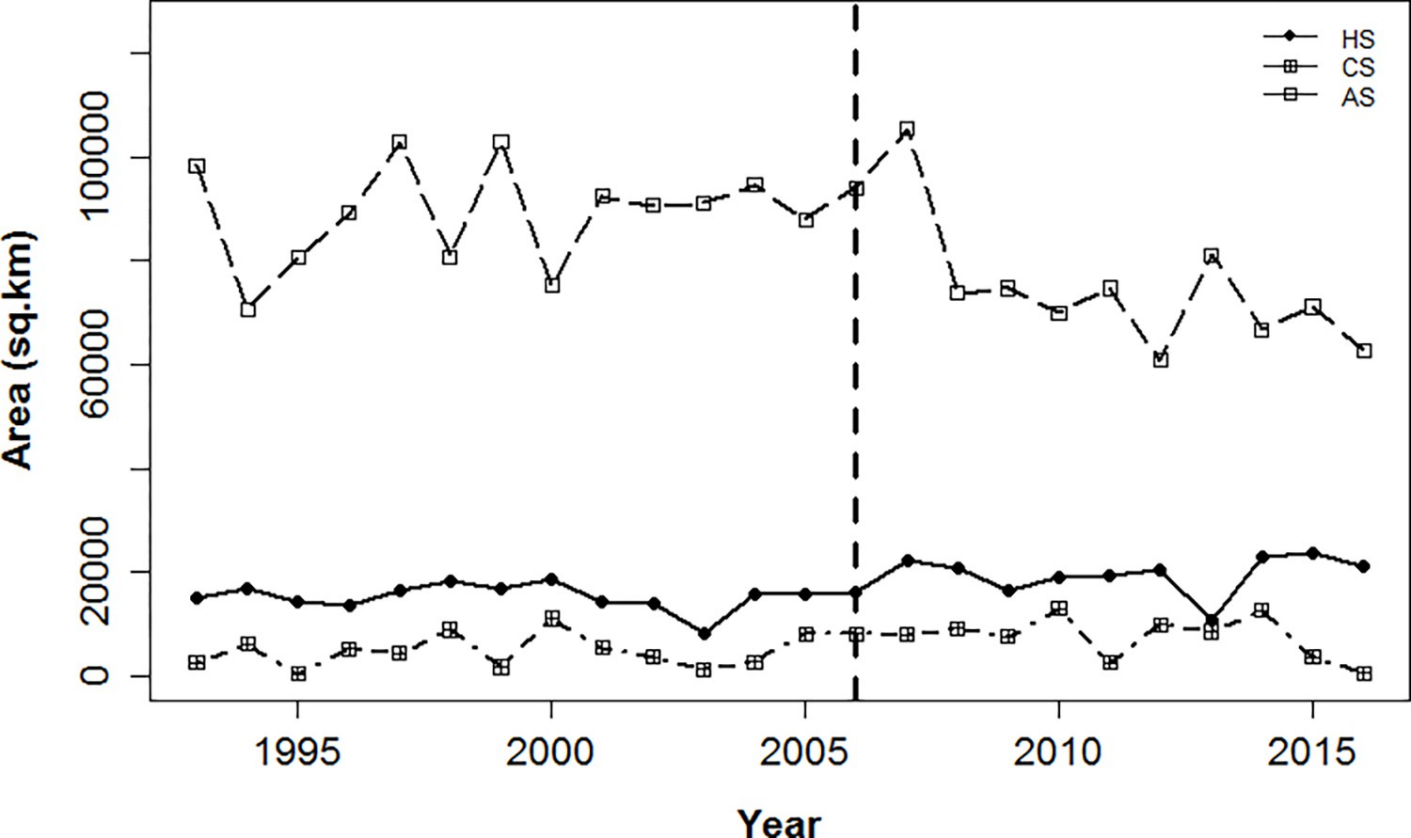
Trend in area (km^2^) under different levels of pheasant productivity over 24-year and 11-year (vertical line) period across HotSpot (HS), ColdSpot (CS) and AverageSpot (AS).

We observed a decline in areas under AverageSpots over both the 11-year (−0.01 km^2^*yr^-1^ ± 0.05) period and the 24-year (−0.02 km^2^*yr^-1^ ± 0.01) period (Fig 5). ColdSpots showed a decreasing trend (−0.03 km^2^*yr^-1^ ± 0.03) over the 11-year period and an increasing trend (0.05 km^2^*yr^-1^ ± 0.03) over the 24-year period (Fig 5). Areas under HotSpots demonstrated a positive trend over both the 11-year (0.02 km^2^*yr^-1^ ± 0.02) and 24-year (0.09 km^2^*yr^-1^ ± 0.03) period (Fig 5).

### Environmental drivers of HotSpots

**Fig 6:**
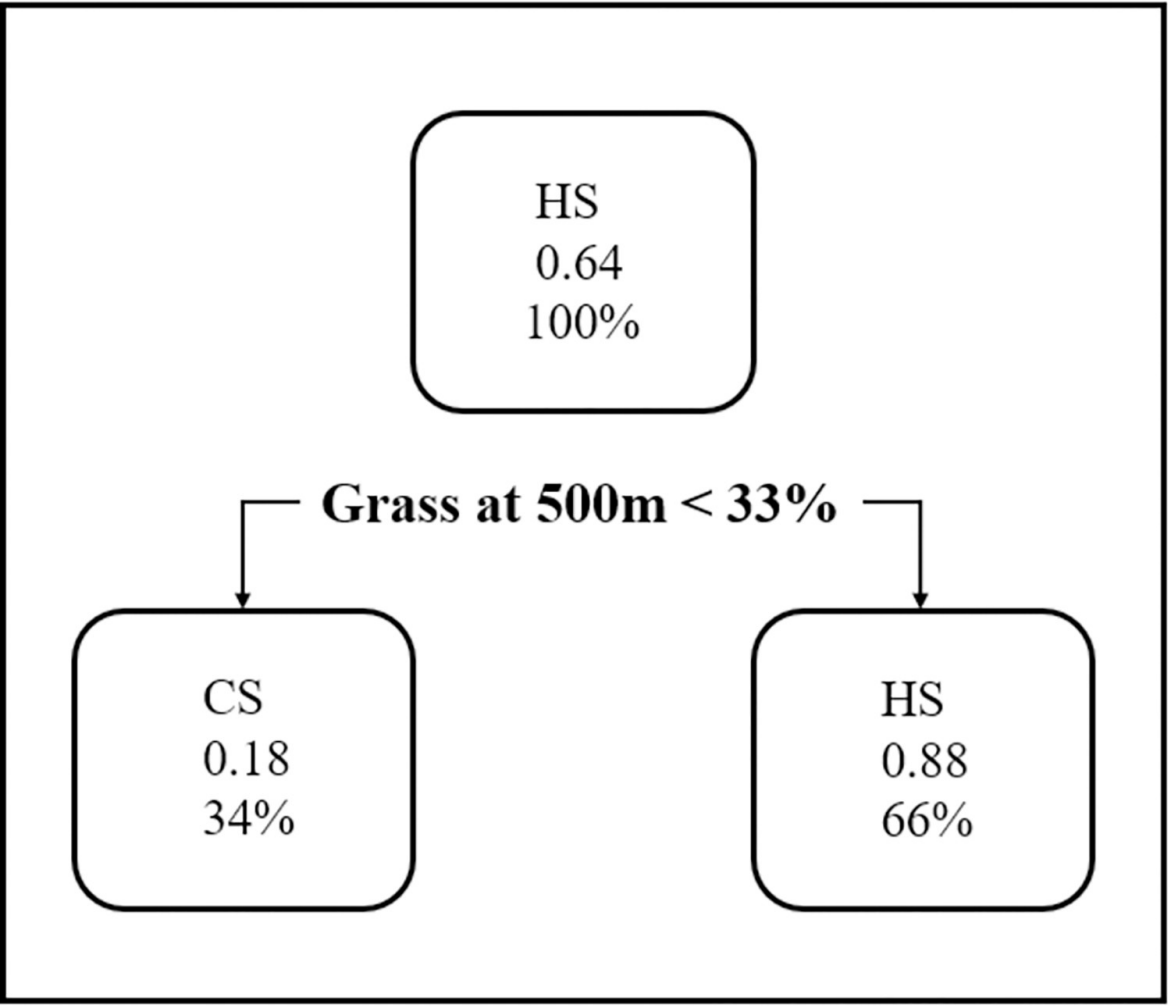
Classification tree model of the influence of landscape attributes on pheasant HotSpots (HS) and Coldspots (CS) at different spatial scales (500 m and 1000 m).

The results of the CART analysis showed grassland area at a 500-m scale to be the only variable influencing HotSpots. HotSpots were predicted for sites with > 33% of the area under grassland at a 500-m scale (Fig 6). The result from confusion matrix was 0.75 which suggests that the model was able to correctly classify a site as either a HotSpot or ColdSpot 75% of the time [47].

## Discussion

Over the 24-year period, pheasant populations in South Dakota have shown a positive population trend, however, during the latter decade, pheasant populations across South Dakota have declined. When we consider spatial variation in pheasant productivity, we observed high rates of decline across HotSpots, ColdSpots, and AverageSpots over the latter 11-year period. We anticipated that HotSpots would occur in areas of high-quality habitat and would support positive pheasant population trajectories [48,49]. Rather, HotSpots also contained declining pheasant populations, albeit at a slower rate than was observed for ColdSpots and Average Spots, suggesting HotSpots had relatively higher suitability for pheasant populations. One potential explanation is that land-use changes over the past decade have incurred an extinction debt upon pheasants and the pheasant population is still responding to the new landscape configuration [50,51]. Similar results have been shown to occur for birds [52], mammals [53], plants [54], and butterflies [51]. This delay in pheasant response can be explained by habitat fragmentation, and habitat loss, both of which degrade habitat quality and therefore affect breeding success, recruitment, and survival [55]. This result could also be attributed to an increase in predator populations in fragmented landscapes [55] which could further negatively impact the pheasant population.

We observed an increase in the HotSpot area with a simultaneous decrease in pheasant abundance and an overall lowering of pheasant numbers required to be a HotSpot over years. This suggests that even though we observed an expansion of the HotSpot area across South Dakota, the overall quality of these Hotspots was declining to be more similar to AverageSpots or ColdSpots. We hypothesize that this could also be a response to increased habitat fragmentation restricting access to resources below a level suitable to sustain pheasant viability [54–56]. Fragmentation may also enhance predation pressure in landscapes by increasing predator abundance and inducing edge effects [57,58]. This further highlights the importance of identifying patches for prioritization in habitat management to deal with a potential extinction debt and avoid future population decline [54,59,60].

Apart from a mosaic of habitat that is necessary to fulfill the life stage requirements for pheasants, pheasant populations are significantly impacted by harsh weather conditions [20,26]. For example, drought is known to limit resources (e.g., concealment and food), which could necessitate increased movements and decreased rates of pheasant survival and reproduction [61], and harsh winter conditions (e.g., high snow depth) which can have severe negative impacts on pheasant survival [62]. The summer of 2012 was one of the harshest droughts in South Dakota history [29]. When coupled with a harsh winter numerous early season blizzards in 2013 [29], 2013–2014 was one of the worst pheasant productivity years across the state (Fig 3). To further exacerbate the situation, one of the largest net losses of grassland area to cultivation occurred from 2012 to 2014 [63]. During this period, we observed that HotSpots exhibited a significant reduction in grassland area and an increase in fragmentation among grassland patches (S1 File). The result of this land-use conversion and climatic stressors combined to result in the greatest *per capita* decline in pheasant counts observed in HotSpots throughout our analysis (Fig 3).

Moreover, we note that in many cases it is not a single stressor that pushes a population past a threshold but a combination of stressors. For example, Sage-grouse (*Centrocercus urophasianus*) in Wyoming were relatively resistant to West Nile Virus or oil and gas fracking, but the combination of both stressors resulted in rapid population decline and in some cases extirpation [64]. Similarly, it appears that in South Dakota the combination of extreme weather events and rampant landscape conversion to cultivation is contributing to the observed pheasant decline.

Despite pheasants being classified by some as habitat generalists, due to their distribution across a wide range of habitats [21,22,26], they are primarily a grassland species and require large tracts of grassland to successfully fledge offspring, and to support adult survival [38,65]. It was not surprising that area under grassland habitat was the main explanatory variable behind predicting an area to be a Hotspot at a small spatial scale. The positive relationship between habitat area and the number of individuals it can support is one of the most important phenomena in ecology and has been frequently used to describe the effects of area loss on species density or their frequency of occurrence [6,66]. Many studies on the impact of fragmented landscapes have demonstrated strong area effects on species abundances and concluded that differences in habitat area is a primary factor determining population persistence [67,68].

We did not find any significant relationship between fragmentation *per se* and pheasant HotSpots, suggesting that pheasants respond more strongly to habitat loss than to fragmentation. This could further be explained by the habitat amount hypothesis (HAH), which suggests that species density increases with the total habitat area in the landscape around sample site [11]. As such, the HAH implies that habitat fragmentation-configuration of patches in landscape-is ultimately non-significant in understanding species density, but they matter only to the extent that they influence the amount of habitat in the local landscape. Similar results, favoring HAH over fragmentation, were observed in many other studies [69–71]. Thus, these results reinforce the HAH and corroborate the idea that fragmentation *per se* has a weak effect on the ecological response of pheasants when the habitat amount is controlled. This study, therefore, helps to inform the debate on the relative importance of habitat amount versus fragmentation *per se* on species abundance [10,72,73]. Our results suggest that conservation efforts for pheasants should focus on habitat preservation and restoration.

These results further contribute to simplification in decision-making policy related to species conservation, since efforts can focus on preventing habitat loss, as well as increasing or maintaining the total habitat amount in the landscape. We suggest that to improve management efficacy and the long-term persistence of populations, managers need to identify ecological factors at multiple scales that enhance, facilitate, or constrain populations. We recommend that managers should focus on preserving and restoring the maximum overall amount of habitat regardless of its configuration. Maintaining habitat amounts by managing habitat patches, large and small, could enhance the benefits of local management practices for pheasants.

In landscape systems where the majority of the land is privately owned, groups of landowners may be incentivized to coordinate efforts at the landscape scale. This process can be expanded to include smaller parcels of public land by developing relationships with neighboring landowners and providing incentives for cooperative conservation agreements among private landowners to facilitate public × private landscape conservation cooperatives. For example, cooperative farming agreements can be utilized whereby private landowners plant crops in a rotation specified by managers to create a landscape mosaic maximally beneficial to pheasants; in turn, landowners may receive either direct payments or tax credits for their participation and adherence to management planting guidelines. This will help in creating more habitats for pheasants in the landscape.

We used pheasants as a model organism to demonstrate the usefulness of the emerging HotSpot analysis for assessing the status of long-monitored species. We demonstrated a novel approach for identifying high productivity areas and factors influencing these areas for a species of management interest at a landscape scale, which could be extended for other species of management and conservation concern. Our use of an emerging HotSpot analysis is the first application of this approach to wildlife count data used to index populations and could be applied to any count surveys that index population abundance. An important feature of this analysis is that it produced an index of relative productivity regardless of annual variation in productivity because even in poor years the highly productive areas were still identified as being more productive relative to other areas of the landscape. Consequently, this analysis identified regions that were relatively more or less productive regardless of annual population performance. This is an important attribute of this analysis as other species of gallinaceous birds have been shown to have high periodicity in annual count data and to respond quickly to environmental conditions [74,75]. Our CART analysis approach also provided a framework for estimating the thresholds of important land-cover types and landscape metrics necessary for sites to be a HotSpot.

## Supporting information

S1 File Distribution and fragmentation indices of pheasant productivity in the landscape. (DOCX)
