# Supplementary material for: Habitat amount or landscape configuration: Emerging HotSpot analysis reveals the importance of habitat amount for a grassland bird in South Dakota
Source: PLoS One. 2022 Sep 26;17(9):e0274808. doi: 10.1371/journal.pone.0274808 (PMC9512187; doi:10.1371/journal.pone.0274808)
Supplement: S1 File — (DOCX) [file pone.0274808.s001.docx]

**Text S1.** Distribution of HotSpot and ColdSpot areas.

The total area that was a HotSpot for some period of time during the 11-year study period was 47,643 km^2^ (~38% of the total study area). This included a core area of 3,512 km^2^ that was consistently a HotSpot for all 11 years (Fig 4a). Bordering this core area to the north and south were 4,153 km^2^ that were HotSpots for 10 years, which were further buffered by another 3,989 km^2^ that were HotSpots for 9 years. In total, ~24% of the study area was a Hotspot for ≥9 years (Fig 4a). The maximum number of years that an area was a ColdSpot was 9 of 11 years as there was no overlap of ColdSpots from 2015 or 2016 to contribute towards the final count. In addition, there was an 8,098 km^2^ area that was a HotSpot in 2007 and ColdSpot for ≥7 years. ColdSpots occupied a total of 20,846 km^2^ (~17 % of study area), of which, there was a 454 km^2^ core that was a ColdSpot for 9 of 11 years. Adjoining this area to the west was 1,214 km^2^ that were a ColdSpot for 8 years, which was further buffered by another 1,343 km^2^ that were a ColdSpot for 7 years. In total, ~14% of the study area was a ColdSpot for ≥7 years (Fig 4b). ColdSpots for the 24-year period had an additional area of 3,938 km^2^ compared to ColdSpots for the 11-year period. Similarly, HotSpots over the 24-year period had an additional area of 7,034 km^2^ compared to HotSpots over the 11-year period.

a)
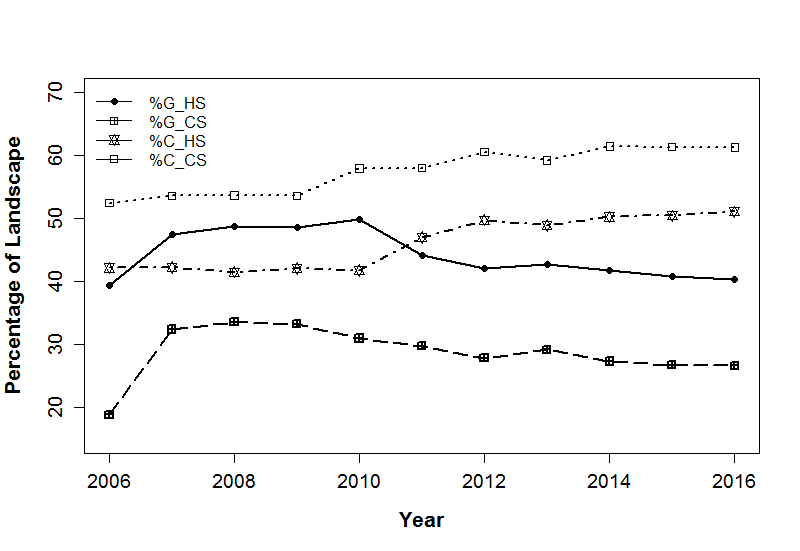


b)
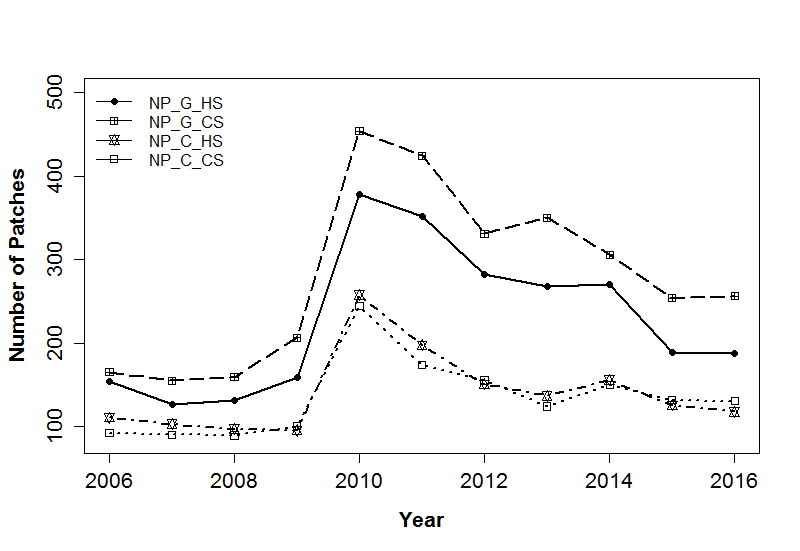


**Fig S1.** Trend in fragmentation indices, a) percentage of landscape under grassland (%G) and cultivation (%C), and b) number of patches in grassland (NP_G) and cultivation (NP_C) across HotSpot (HS) and ColdSpot (CS) over 11-year period.
